# Strengthening behavior of carbon/metal nanocomposites

**DOI:** 10.1038/srep16114

**Published:** 2015-11-06

**Authors:** S. E. Shin, H. J. Choi, J. Y. Hwang, D. H. Bae

**Affiliations:** 1 Department of Materials Science and Engineering, Yonsei University, Seoul 120-749, Korea; 2 School of Advanced Materials Engineering, Kookmin University, Seoul 136-702, Korea; 3 Carbon Convergence Materials Research Center, Korea Institute of Science and Technology (KIST), Wanju-gun, Jeonbuk 565-905, Korea

## Abstract

Nanocomposites reinforced with nano-scale reinforcements exhibit excellent mechanical properties with low volume fraction of the reinforcement. For instance, only an addition of 0.7 vol.% few-layer graphene (FLG) into the pure titanium shows strength of ~1.5 GPa, obviously much superior to that of the monolithic titanium. The strengthening efficiency of composites is determined by several factors such as reinforcement geometrical/spatial characteristics and interfacial features between the matrix and the reinforcement. For the metal-matrix nanocomposites (MMNCs), since the nano-scale reinforcement has significantly high specific surface area, interfacial feature is more important and has to be clearly evaluated in understanding property of MMNCs. Although many researchers suggested the theoretical work using continuum mechanics in order to estimate the mechanical properties of the metallic composites, a clear determination has yet not to be proven by systematic experimental works. Here, we provide a new model to predict strength and stiffness of MMNCs based on quantitative analysis of efficiency parameters in which interface feature is strongly emphasized. To validate the model, we select multi-walled carbon nanotube (MWCNT) and FLG for reinforcement, and titanium (Ti) and aluminum (Al) for the matrix to modify bonding strength and specific surface area in the MMNCs.

Among the composite materials, the endeavor to develop light-weight metal matrix composites (MMCs) with excellent performance has been conducted for structural applications. MMCs are known for their ability to retain good properties of metals without compromising strength and stiffness limitations of monolithic metals^1^,^2^,^3^,^4^,^5^,^6^,^7^,^8^,^9^,^10^. Weight reduction through the development of new structural composites with excellent mechanical performances is an effective strategy to improve energy efficiency of vehicles. The use of light metals, such as Ti and Al, is common within the automotive and aerospace industries^11^,^12^. Nano-carbon (nano-C) materials have been considered to be used as reinforcing agents for the light metal matrix composites. They are allotropes of carbon consisting of large molecules where carbon atoms are arranged in the forms of sphere (i.e. fullerene), tube (i.e., CNTs) and sheet (i.e. graphene). The interest of reinforcing MMNCs with nano-C materials has grown rapidly due to their excellent mechanical properties and chemical stabilities^13^,^14^,^15^,^16^,^17^.

The strengthening of MMNC by nano-C materials can be optimized when the matrix transfer load to the reinforcement effectively. Generally, the reinforcement carries higher amount of load than the matrix^4^. In addition to the mechanical properties of the reinforcement, theoretical works have reported that the geometry of the reinforcement and its interface to the matrix could also play an important role in strengthening^18^,^19^,^20^,^21^. However, strengthening models to prove interfacial bonding linking the composite strength or stiffness is not attended. Here, we introduce meal/nano-C composites that exhibit world-record strength and stiffness, and are fabricated from cheap carbon materials using an industrially favorable route. We produced a set of composites with two different matrices (i.e., Ti and Al) and two types of reinforcements (i.e., MWCNTs and FLG) in order to study the effects of interfacial bonding and geometry of reinforcement on the mechanical properties of nano-structured MMCs.

Simulations with density-function theory (DFT) calculated the energetically favorable adsorption site in graphitic structure for certain metal elements. The equivalent bonding strength and the distance between graphene and metal were determined from the simulations as well^22^,^23^. As depicted in Fig. 1a, the carbon (C) atoms are held together by strong covalent bonds in the basal graphitic plane. The remaining *p*_z_-orbital of carbons allows them to bond with metals out of the plane. Non-transition metals (e.g. Al) form weak secondary bonds with graphene since they lack *d*-sub shell with very limited affinity with carbon. Transition metals (e.g. Ti), on the other hand, have unfilled *d*-orbitals where *d*-electrons may form ionic bonds with the dangling branches of carbon atoms in graphene. Calculations reveal that the overall bonding strength between the basal plane of Ti and a single plane of graphene was calculated to about five times higher than that between Al and carbon^21^.

The carbon and metal matrix composites for both Ti and Al were observed with high-resolution transmission electron microscopy (HRTEM) images and electron energy loss spectroscopy (EELS) analysis. Atomic-scale observation of the composite in powder-processed MMNCs can provide considerable information on the interface structure. The interface between FLG and Al matrix (Fig. 1b) appears different from that between FLG and Ti matrix (Fig. 1c). The HRTEM image of the FLG/Al composite clearly showed typical lattice fringes of a single plane of graphite with an inter-layer spacing of ~0.34 nm that confirms the presence of FLG (thickness: ~5 nm). This spacing is not observed in the FLG/Ti composite. Furthermore, the interface between Al matrix and FLG is obvious whereas the interface between Ti matrix and FLG appears in a stark contrast. Nonetheless, the Raman spectra confirm the presence of FLG for both composites (Supplementary Fig. S4). Figure 1c shows HRTEM image of the FLG/Ti composite where carbon is detected using bright and dark field images (Supplementary Fig. S5). The interference between FLG and Ti caused FLG to possess Moire fringes. It is hypothesized that Ti–C ionic bonds, partially formed between Ti and FLG, results in a heterogeneous arrangement of lattices near the FLG surfaces. The different bonding features between these two composites are clearly demonstrated in EELS analysis. Slight variations in EELS spectra along the detection points (from (i) to (iii) in the HRTEM images) indicate the presence of partly balanced incomplete metal-carbon bonds for both composites. Typically, C peaks at 285 eV near FLG while Al peaks at 1563 eV near Al matrix. Any types of Al-C ionic bonds (Al and C peaks at 73.4 and 282.2 eV, respectively) are not detected around the interface. The thermo-mechanical condition to produce the FLG/Al composite was not sufficient to form Al carbides along the interface^20^. Thus, clean interface was formed by means of micromechanical interlocking associated with the FLG and Al matrix. Transition metal, on the other hand, is strongly electrophilic and reactive to form Ti-C ionic bonds. Thereby, Ti provides the ionic-bonded C-Ti species at a relatively notable intensity of 458 eV in FLG as well as in Ti matrix.

High-resolution XPS analysis, shown in Fig. 2, compares the bonding characteristics of MWCNTs/Ti, and FLG/Ti composites. The contributions of Ti (2p_1/2_) and Ti (2p_3/2_) spin-orbital splitting photoelectrons to Ti^4+^ are identified at excitation energies of 463.5 and 457 eV. Moreover, carbon bonds with the π-π^*^ shake-up, sp^2^, and sp^3^ hybridization are detected at binding energies of 286.3, 283.4, and 284.1 eV, respectively. The binding energy of Ti-C bonds exists at 282.8 and 454.5 eV for C 1 s and Ti 2p, respectively^24^,^25^. Supplementary Table. S1 summarizes the binding energy and peak area of each peak in the XPS spectra. Furthermore, Supplementary Table. S2 calculates the portion of Ti-C bonds in the MWCNTs/Ti and FLG/Ti composites. Each composites are normalized with the reinforcement volume (i.e., 1 vol.%). FLG/Ti composites show twice as much volume fraction of Ti–C bonds than MWCNTs/Ti composite. The chance of forming Ti–C ionic bonds on FLG may be higher than MWCNTs due to its superior specific surface area.

Mechanical properties of nano-C reinforced Ti and Al composites, as a function of the reinforcement volume fraction (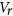), are displayed in Fig. 3a,b. The original compressive stress-strain curves of the composites are shown in Supplementary Fig. S6. The elastic modulus (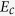) and yield stress (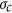) of each composite increase with the volume fraction of reinforcements, although the slope of the increment starts to drastically decrease when the content of FLG and MWCNT is over 1 and 7 vol %, respectively. High volumes of MWCNT and FLG added within the metal matrix tend to agglomerate into clusters during the composite processing because of their large surface areas (as shown in Supplementary Fig. S7). These carbon aggregates may restrict consolidation of the composite powder, generating pores or carbides in the final composite, thereby deteriorating the mechanical properties. On the other hand, the increment of 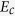 and 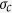 are notably varied by the type of matrix and reinforcement used; FLG and Ti matrix show better strengthening efficiency over MWCNTs and Al matrix, respectively. Since the composites contain discontinuous reinforcements, primary deformation occurs in the matrix when the composites are loaded. The strained matrix transfers load to the reinforcements by means of shear stresses that generate along the matrix/reinforcement interface. The mechanism behind load transfer is governed by three important parameters: i) interfacial bonding between the reinforcements and the matrix (the bonding factor,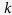); ii) the aspect ratio and the surface-to-volume ratio of the reinforcement (the geometry factor,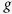); and iii) the average angle between the loading direction and the reinforcement axis (the alignment factor,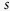). We modified the rule of mixture with these three parameters, in order to emphasize their effects on the load transfer behavior, as expressed in Eq. (1) and (2); details in derivation of the formula are present in Supplementary Information:


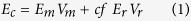



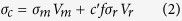


where *E*_*m*_ and *E*_*r*_ are the elastic modulus of matrix and the reinforcement, *V*_*m*_ and *V*_*r*_ are the volume fraction of the matrix and reinforcement, *σ*_*m*_ and *σ*_*r*_ are the yield strength of the matrix and the reinforcement, respectively. *c* and 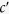 are empirical constants for *E*_*c*_ and 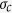, respectively. An efficiency factor *f* is given by (Table 1):


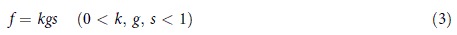


In Figure 3c,d, a differing interfacial bonding of Ti-C and Al-C is well contrasted with Ti and Al matrix with plots of normalized *E*_*c*_ and *σ*_*c*_ as a function of the surface area per unit volume (*gV*_*r*_). The surface area per unit volume, equivalent quantity of reinforcements per arbitrary region (further details on the calculation each parameters are provided in Supplementary Information), therefore, relatively small amount of FLG in comparison with MWCNTs can be balanced using *gV*_*r*_. Accordingly, normalized *E*_*c*_ and *σ*_*c*_ by *gV*_*r*_ are expressed as 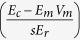 and 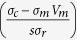, respectively. For the same matrix, the increment of normalized *E*_*c*_ and *σ*_*c*_ is similar for both reinforcements, which data values lie on the same line and *E*_*c*_ and *σ*_*c*_ are proportionally increases with *gV*_*r*_, whereas, slopes for different matrix are distinguished according to bonding strength. Moreover, the geometry effect alone cannot cause the spectral shape changes of metal-C bonding, in other words bonding character is not responsible for reinforcement geometry^26^. *E*_*c*_ and *σ*_*c*_ are further normalized by bonding factor using the Eqs (1) and (2), which expressed as 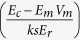 and 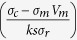, respectively, and plotted as displayed in Fig. 3e,f, respectively. According to plots, we find values of 33.8 and 14.4 for the *c* (for *E*_*c*_) and 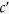 (for *σ*_*c*_), respectively.

Here, we show the numerically and experimentally demonstrated the contribution of factors to strengthening behavior of MMNCs reinforced by nano-C. Based on our results, when nano-C reinforcements are separately dispersed in metals, we can predict mechanical properties as a function of specific surface area when the bonding strength between the carbon and the metal is determined. Also, a combination of tight interfacial bonding and large specific surface area in the reinforcement/matrix interface would possess an efficient load transfer and enhanced properties of the composite. We explain the importance behind the dimensions and the interfacial properties of reinforcement that determine the performance of composites. The results provide a guideline for the design of MMNCs with diverse reinforcements using the reliable model to predict mechanical properties of MMNCs.

## Methods

### Composite powder preparation

Both Ti and Al-based composites reinforced by nano-C (MWCNTs and FLG) were produced by powder metallurgy. FLG is pre-milled using a planetary mill (Fritsch Co. Ltd., Pulverisette 5, Germany) before mechanical milling. FLG from graphite flakes (6−8 nm thickness and 120−150 m^2^/g typical specific surface area) were mechanically exfoliated by planetary ball milling with isopropyl alcohol ((CH3)2CHOH, IPA). A stainless steel bowl (500 mL) was charged with graphite flakes (2 g) and stainless steel balls (~5 mm diameter, 30 g) at a ball-to-powder weight ratio of 15:1, together with 50 ml of IPA. Planetary milling was performed at a rotation speed of 200 RPM for 1 h; it was paused for 75 min after every 15-min milling to maintain ambient processing temperature without any process control agent. Afterwards, the IPA was evaporated and dried at 150 ^o^C for 3 h. Graphite flakes are supposed to be exfoliated by shear forces on contact between powder and balls during milling. Due to the weak van der Waals-like coupling between graphite layers, the graphene sheets in graphite can slide easily with respect to one another. For FLG/Ti composites, exfoliated graphite flakes and Ti powders were mixed without a process control agent using a planetary mill with a rotation speed of 100 RPM for 3 h; it was paused for 15 min after every 15-min milling to maintain processing temperature as the room temperature. For FLG/Al composites, firstly, **e**xfoliated graphite flakes and Al powders were mixed with a process control agent of 1 wt.% stearic acid using a planetary mill with a rotation speed of 100 RPM for 3 h; it was paused for 15 min after every 15-min milling to maintain processing temperature as the room temperature. For both FLG/Ti, Al composites, to distribute FLG in Ti (Al) powders, the mixed powder was high energy ball-milled in an attrition mill at 500 RPM for 6 h under a purified argon atmosphere at a ball-to-powder weight ratio of 15:1, respectively. On the other hand, for MWCNTs/Ti, Al composites, to distribute MWCNTs in Ti (Al) powders, the mixed powder was high energy ball-milled in an attrition mill at 500 RPM for 6 h under a purified argon atmosphere at a ball-to-powder weight ratio of 15:1, respectively.

### Composite Fabrication

Hot-pressing was carried out to consolidate a variety of ball-milled composite powders by varying the volume fraction of the reinforcements. Prior to pressing, the ball-milled powder was put in a stainless steel die with a diameter of 30 mm, which was surrounded by graphite foil. The punch and plate were sprayed with boron nitride, which was used as a lubricant to minimize the effect of friction. The powder was then pressed in the mold at 140 MPa at 450 °C for 1 h (Al-based composites) and at 570 °C for 1 h (Ti-based composites). After pressing, the graphite foil was easily peeled off. As the MWCNTs and FLG were deeply embedded in the metal powders, they were not expected to significantly interrupt the consolidation of powder during hot pressing, providing a fully dense composite compact.

### Mechanical property testing

The compressive properties of the specimens were evaluated using an Instron-type machine under a constant crosshead speed condition of an initial strain rate of 1 × 10^−4^ s^−1^ at room temperature. Rectangular specimens with 2:1 ratio of height-to-width were prepared for compression tests and evaluated. Two tungsten carbide plates were used to sandwich the compression specimens, and a sprayed film of boron nitride was used as a lubricant to minimize the effect of friction. Nanoindentation tests on the specimens were performed using a commercial nanohardness tester (Nanoindenter XP, MTS) equipped with a Berkovich indenter. In each test, the indenter was driven into the sample surface (loading half-cycle) at a rate of 10 nm/sec^−1^ and the peak load ranges from 20 mN to 200 mN.

### Characterization techniques

The microstructure of the composites was observed using a high-resolution transmission electron microscope (HRTEM, Titan TM 80–300, FEI). Thin foil specimens from the sheets were carefully prepared by an ion-beam milling method (Gatan, Model 600, Oxford, UK). Electron energy loss spectroscopy (EELS) data were collected using an incident e-beam of 1200 eV, a 0.5 eV per step and resolution of 2 eV, measured from the full-width at half-maximum (FWHM) of backscattered electrons. For the X-ray photoelectron spectroscopy (XPS, K-alpha, Themo VG, UK) measurements, after exciting the films by the Al Kα line (1486.6 eV) and completed at resolution of 50 and 0.1 eV energy steps. The energy scale was measured in the Ag 3d_5/2_.

## Additional Information

**How to cite this article**: Shin, S. E. *et al.* Strengthening behavior of carbon/metal nanocomposites. *Sci. Rep.* **5**, 16114; doi: 10.1038/srep16114 (2015).

## Supplementary Material

Supplementary Information

## Figures and Tables

**Figure 1:**
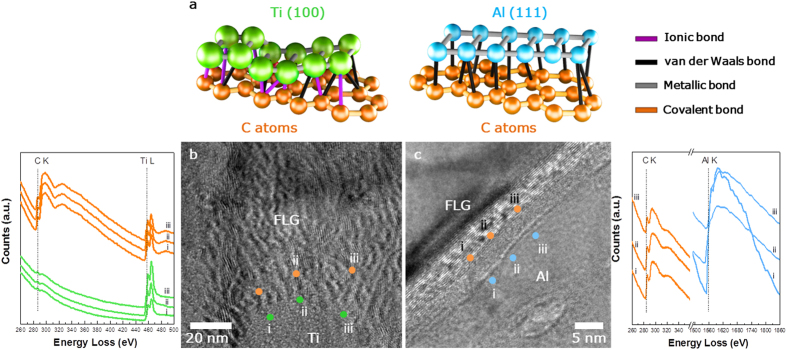
HRTEM and EELS characterization. (**a**) Schematic of bonding features for FLG/Al and FLG/Ti composites. Four possible variety of bond are signified different colors; purple, black, blue and orange colors are corresponding to Ionic, van der Waals and covalent bonds, respectively. High-resolution TEM images showing the microstructure of (**b**) FLG/Ti and (**c**) FLG/Al composites. Corresponding EELS spectra taken from the FLG/Ti and FLG/Al composites indicate the existence/nonexistence of metal-carbon bond at the interface between metal matrix and FLG. An EELS line scan analysis of the structure from for the C–K, Ti-L and Al–K edges, confirms that the atoms consists of the composites.

**Figure 2:**
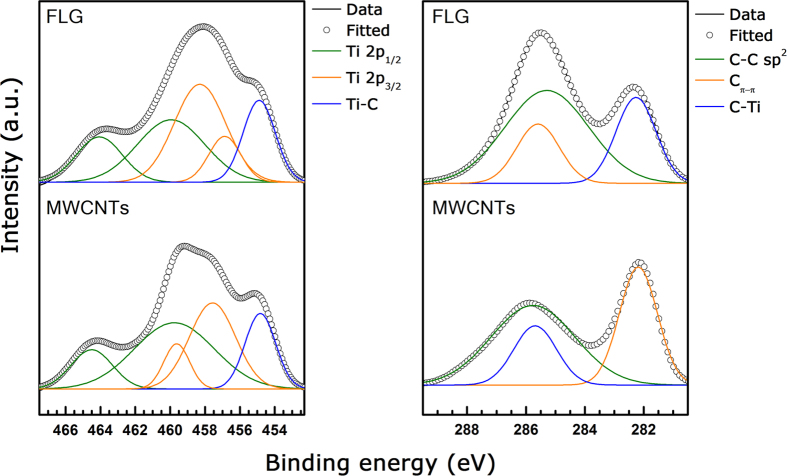
XPS spectra for the C/Ti composites. Ti 2p and C 1s. Full lines are measured data and open circles are mathematical fit. For the Ti-based composites, olive, orange and blue lines correspond to the Ti 2p_1/2_, Ti 2p_3/2_ and Ti-C bonds, respectively. Al-based composites, olive, orange, magenta and blue lines correspond to the C-C sp^2^, C-C π–π, and C-Ti bonds, respectively.

**Figure 3:**
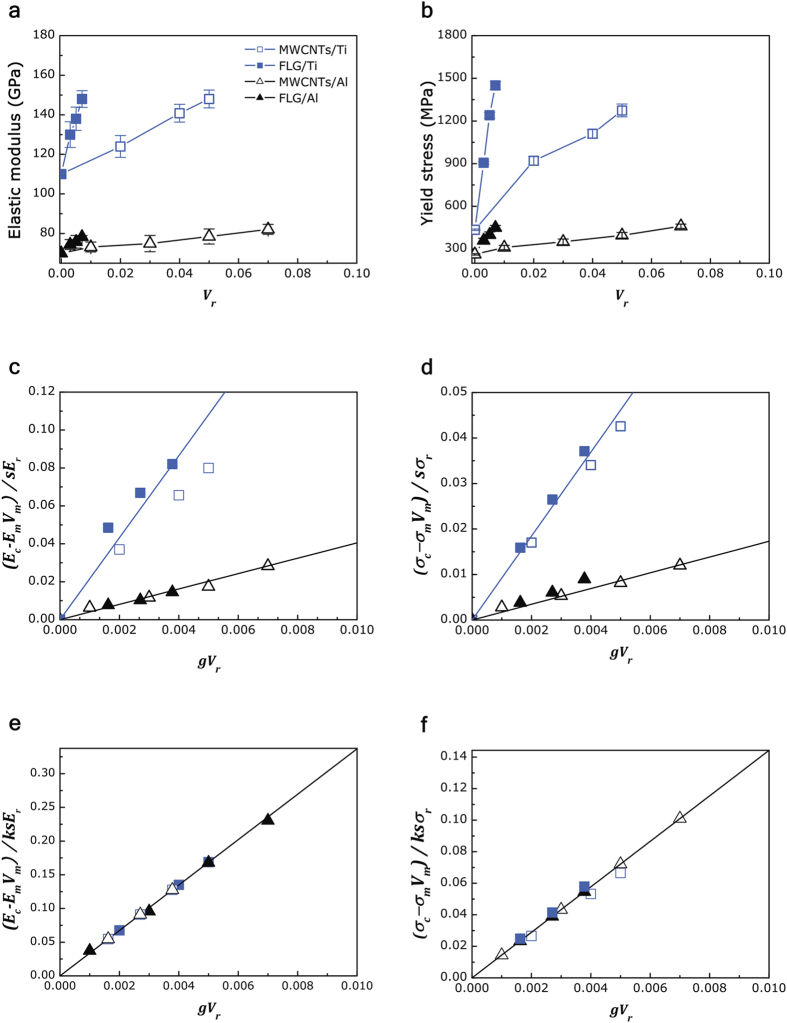
Mechanical behavior of the composites. (**a**,**b**) Measured value of (**a**) elastic modulus (*E*_*c*_) and (**b**) compressive yield stress (*σ*_*c*_) of composites as a function of the volume fraction of the reinforcements (*V*_*r*_). Left column: normalized 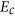. Right column: normalized *σ*_*c*_. (**c**,**d**) normalized *E*_*c*_ (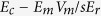) and normalized *σ*_*c*_ (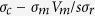) as surface area per unit volume (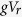). (**e**,**f**) normalized 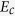 (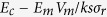) and normalized 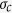 (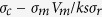) as bonding factor (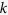).

**Table 1:** Elastic modulus and yield stress of the C/Al and C/Ti composites.

Matrix		Aluminum							Titanium					
Reinforcement		MWCNT				FLG			MWCNT			FLG		
vol.%		1	3	5	7	0.3	0.5	0.7	2	4	5	0.3	0.5	0.7
Parameter	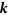	0.12							0.64					
	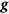	0.10				0.54			0.10			0.54		
	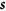	0.60												
Constant	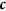	33.8												
Elastic modulus (GPa)	Calculated	72.0	74.8	78.6	81.7	73.7	76.2	78.7	133.7	157.4	169.3	130.6	144.4	158.2
	Measured	73.0	75.0	78.0	82.1	74	75	78	124	140	148.5	130	138	148
Slope 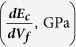	Calculated	166				1243			1185			6886		
	Measured	161				1094			770			5365		
Constant	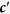	14.4												
Yield stress (MPa)	Calculated	291	348	405	462	312	345	378	733	1031	1179	720	910	1100
	Measured	310	350	396	460	360	400	450	933	1174	1274	906	1240	1450
Slope 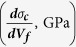	Calculated	2.80				16.6			14.8			95.9		
	Measured	2.70				26.5			16.5			147.7		
