# Supplementary material for: Strengthening behavior of carbon/metal nanocomposites
Source: Sci Rep. 2015 Nov 6;5:16114. doi: 10.1038/srep16114 (PMC4635460; doi:10.1038/srep16114)
Supplement: Supplementary Information [file srep16114-s1.doc]

**Strengthening behavior of nano-carbon/metal nanocomposites**

S.E. Shin^1^, H.J. Choi^2^, J.Y. Hwang^3^ and D.H. Bae1[[1]](#footnote-2)*

^1^Department of Materials Science and Engineering, Yonsei University, Seoul 120-749, Korea

^2^School of Advanced Materials Engineering, Kookmin University, Seoul 136-702, Korea

^3^Carbon Convergence Materials Research Center, Korea Institute of Science and Technology (KIST), Wanju-gun, Jeonbuk 565-905, Korea

**A. Synthesis of C/Ti, C/Al composite powders**

Morphology of the ball-milled powder was observed using scanning electron microscopy (SEM, JSM‒7001F, JEOL). Several different milling conditions of the powder revealed different results. Graphite flakes were also exfoliated using a planetary mill. 2 g of graphite flakes and 30 g of stainless steel balls were charged into a stainless steel bowl (250 ml) at a ball-to-powder weight ratio of 15:1. 50 ml of IPA was used as a PCA. Planetary milling was performed at a rotation speed of 200 RPM for 1 h; it was paused for 75 min after every 15-min milling for 4 cycles to maintain the ambient processing temperature. During the wet grinding process, the IPA solution helps the sliding motion from graphite to graphene without severely damaging the initial molecular structure. The IPA was evaporated at 150 ^o^C for 3 h after fullerene soot or graphite flakes were wet milled. Figs. S1a–c display scanning electron microscope (SEM) images of pristine fullerene aggregates (Fig. S1a), multi-walled carbon nanotubes (MWCNT, Fig. S1a), and graphite flakes (Fig. S1b). Figs. S1c shows SEM image of exfoliated graphite flakes (also known as few–layer graphene (FLG)); it has undergone wet milling process were taken.

**Fig. S1.** SEM images of as-received **(a)** MWCNT, **(b)** graphite flakes and **(c)** exfoliated FLG.

Exfoliated FLGs are mixed with Ti or Al powder (99.5% in purity and <150 μm in the average diameter) using a planetary mill. Planetary milling process was carried out with a rotation speed of 100 RPM for 1 h; it was paused for 75 min after every 15-min milling for 4 cycles to maintain the ambient processing temperature. For Ti-based composite powder, no PCA was used throughout the process, whereas for Al-based composite powder, 1 wt. % stearic acid (CH3(CH2)16COOH) was added as a PCA; this would prevent excessive cold welding of Al powder. Furthermore, the planetary–milled composite powders were ball-milled using an attrition mill at 500 RPM for 6 h in a purified argon atmosphere at room temperature. The morphology of Ti-based composite powders is displayed in Fig. S2. Figs. S2b and d are attrition–milled MWCNT/Ti, and FLG/Ti powders respectively. Magnified images of the rectangles marked in Figs. S2b and d are shown in Figs. S2c and e, respectively. Reinforcements are not observed on the surface of both powders. Therefore, reinforcements are supposed to be embedded and dispersed inside the Ti and Al powder. Likewise, figure S3 exhibits the morphology of pure Al (Fig. S3a) and attrition-milled Al-based composite powders (Figs. S3b–g). Figs. S3b and d are attrition–milled MWCNT/Al and FLG/Al powders, respectively. Figs. S3c and e are the magnified images of the rectangles marked in Figs. S3b and d, respectively.

**(a)**


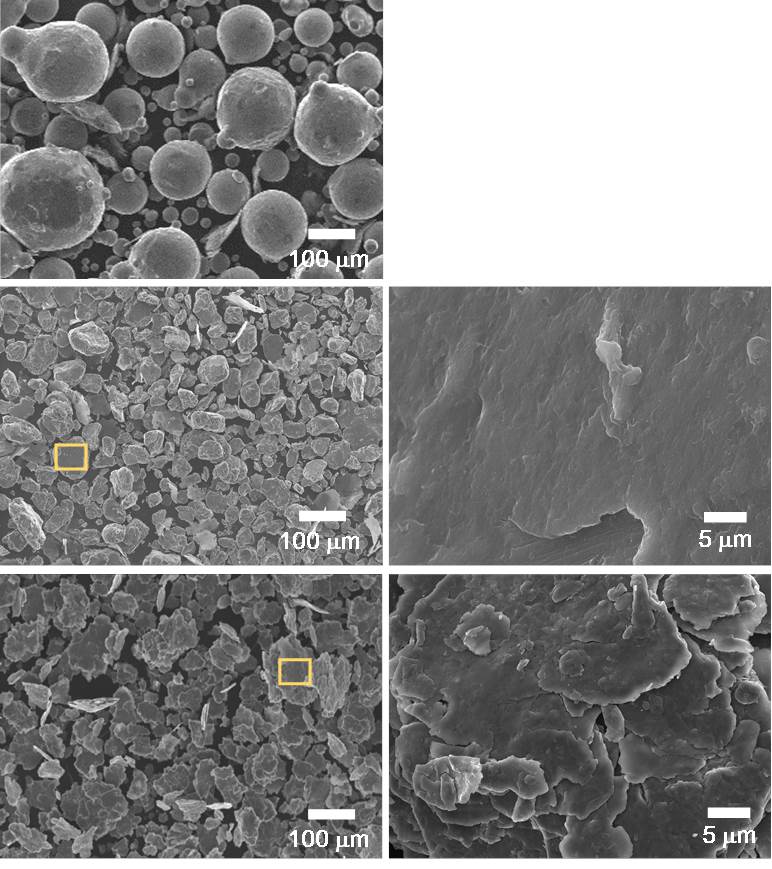


**(b)**

**(c)**

**(d)**

**(e)**

**Fig. S2.** SEM images of **(a)** Pure Ti powder, **(b)** MWCNT/Ti and **(d)** FLG/Ti composite powders. (c) and (e) are magnified images of (b) and (d), respectively.


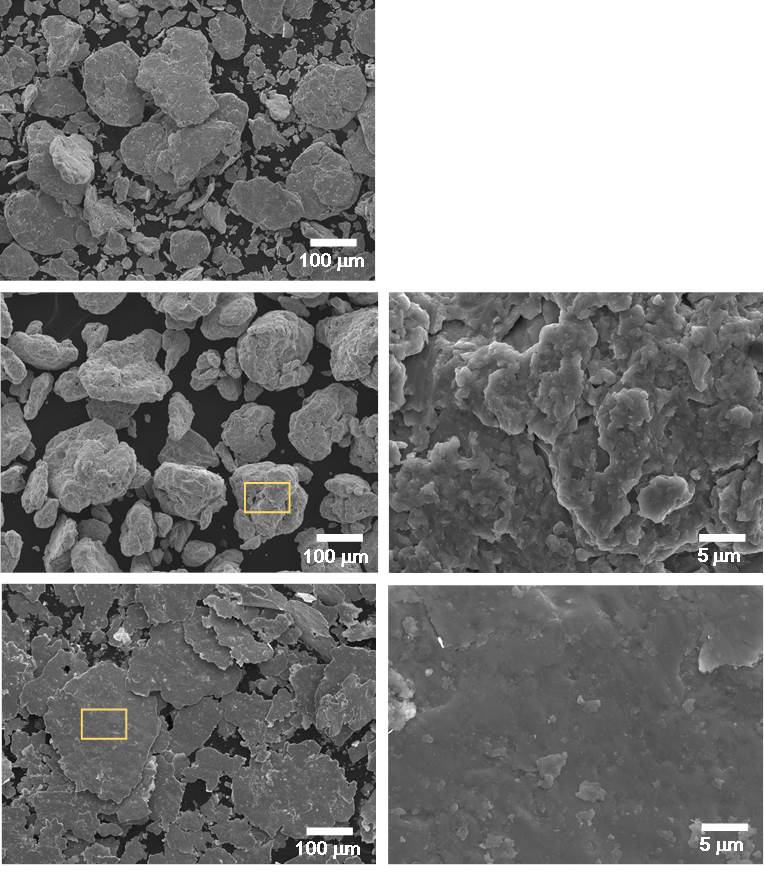


**(a)**

**(b)**

**(c)**

**(d)**

**(e)**

**Fig. S3.** SEM images of **(a)** Pure Al powder, **(b)** MWCNT/Al and **(d)** FLG/Al composite powders. (**c**) and (**e**) are magnified images of (b) and (d), respectively.

The ball-milled composite powder was consolidated by hot-pressing, which is an industrially favorable route. Prior to pressing, the ball-milled powder was put in a stainless steel die with a diameter of 30 mm surrounded by graphite foil. The punch and plate were sprayed with boron nitride to lubricant and minimize the friction between the mold and the samples. The powder was pressed in the mold under a constant pressure of 140 MPa at 500 °C (Al-based composites) and at 570 ^o^C (Ti-based composites) for 1 h. After pressing, the graphite foil was peeled off. Since the reinforcements were deeply embedded in the powder, they provide a fully dense composite pallet without significantly interrupting the consolidation of the powder.

**B. Microstructures of C/Ti, C/Al composites**

The variation in the morphology of the ball-milled powder was observed with SEM. The structure of the reinforcements during the annealing was also investigated by Raman spectroscopy using a Jobin-Yvon microspectrometer (LabRam HR, Jobin-Yvon Co. Ltd., France). The spectra were collected under the ambient condition using the 514.5-nm line of argon-ion laser. The microstructure of the composites was observed using a high-resolution transmission electron microscope (HRTEM, Titan TM 80-300, FEI). Thin foil specimens from the sheets were carefully prepared by an ion-beam milling method (Gatan, Model 600, Oxford, UK). Energy dispersive spectroscopy (EDS) attached to the HRTEM was used to verify the phase in the composites at accelerating voltage of 300 kV. After exciting the films by the Al Kα line (1486.6 eV), X-ray photoelectron spectroscopy (XPS, K-alpha, Themo VG, UK) was completed at 50 eV energy resolution with 0.1 eV energy step. The energy scale was measured in the Ag 3d_5/2_.

Fig. S4 shows Raman spectra of initial MWCNT and graphite flakes. It also shows as-pressed MWCNT/Ti, Al and FLG/Ti, Al composites. Typical G– and D– peaks are observed for all samples, indicating the presence of graphitic materials after severe thermo–mechanical processes. The initial MWCNT sample exhibits D (defect) and G (graphite) peaks at around 1350 and 1575 cm^-1^, respectively [1]. Also, D-peak, G-peak, and 2D band (shape of the second-order Raman bands) of graphitic carbons are detected in the initial graphite powder at 1359, 1603, and 2727 cm^-1^, respectively [2, 3]. G-peak shifts to a higher wave number for both MWCNT/Ti and Al composites (i.e., from 1575 to 1609 cm^-1^). The peak shift generally occurs when MWCNT are under a compressive state induced by the high impact energy of milling media on the powders during the milling process (peak shift marked by red dotted line for Al-based composites and blue dotted line for Ti-based composites) [4]. Also, FLG/Ti and Al composites show G-bands shifting toward lower values as milling proceeds. It is presumed that the surface of graphite powder strongly interact with isopropyl alcohol. Thus, the functionalized surface may generate a certain amount of in-plane internal stresses and weaker C‒C bonds [5]. The peaks shift back toward their expected position after attrition and planetary milling with aluminum powder. FLG dispersed inside aluminum may not interact with other material. Thus, the residual stresses could be released. The ratio between the intensities of the D and G bands (i.e. I_D_/I_G_) is considered to be the ratio of structural defects and domain size in graphitic materials [6]. The I_D_/I_G_ ratio increases significantly after planetary milling with isopropyl alcohol. It further saturates after attrition milling and hot-rolling processes due to partial destruction of the molecular structure of FLG. The down-shifted 2D band (from 2727 cm^-1^ to 2678 cm^-1^), which is related to the crystalline graphitic structure, arises due to a reduction of the number of graphite layers during the ball milling process [7]. The 2D band of FLG/Ti and FLG/Al composites presents a sharp and symmetric peak shape; it was comparable with those of FLGs (<5 layers) [8]. On the other hand, the 2D band of graphite is fitted in two peaks, which is typically observed in 10‒20 layers of graphene [8].

**Fig. S4.** Raman spectrum of FLG reinforced MWCNT reinforced composites.

**
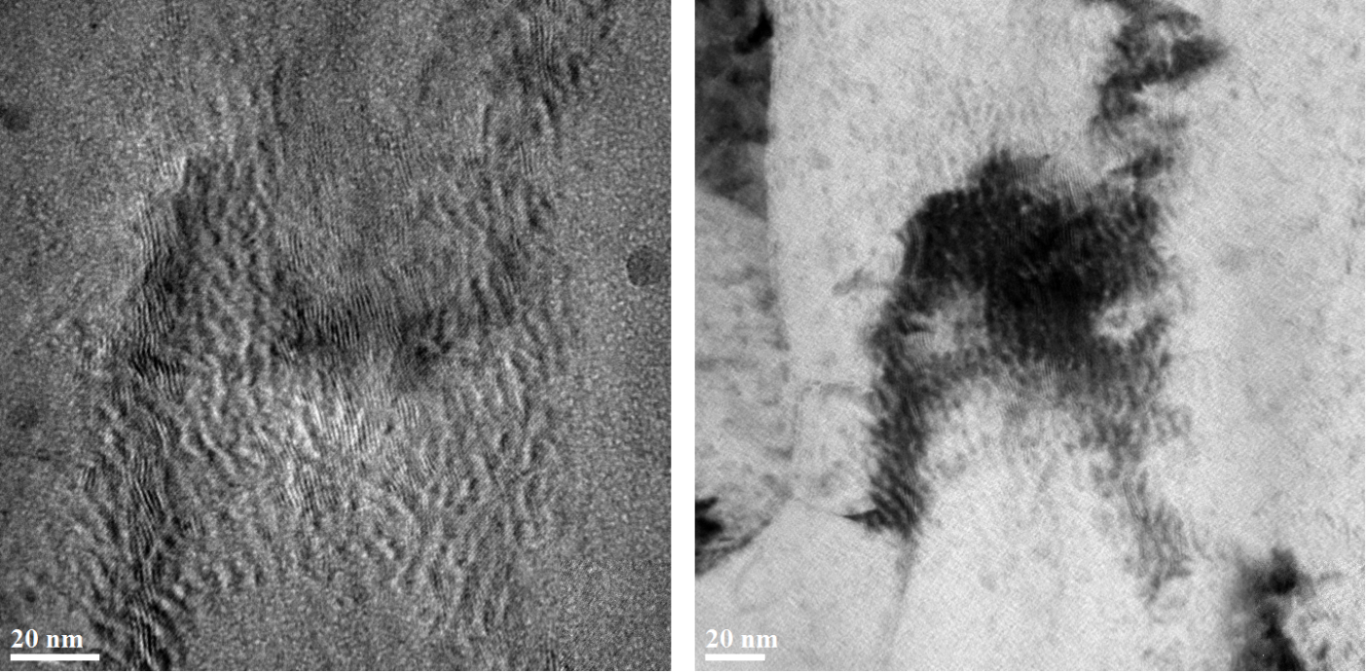
**

**(a)**

**x**

**x**

**(b)**

**(c)**

**(d)**

|  | **wt%** | **at%** |
| --- | --- | --- |
| **Ti** | 99.85 | 91.26 |
| **C** | 2.40 | 8.74 |
| **Totals** | 100.0 | 100.0 |

**Fig. S5.** HRTEM images of FLG/Ti composites. **(a)** Bright and **(b)** dark field images (FLG is marked by an arrow), and **(c)**, **(d)** EDS analysis of the composite.

HRTEM images of the FLG/Ti composite using bright and dark field images are shown in Fig. S5 (FLG is marked by an arrow). Wavy and rumpled morphology of FLG is clearly shown in the bright and the dark field images. EDS analysis in Fig. S5c and d confirms the presence of carbon atoms that is presumably associated with FLG.

The effect of the reinforcement type on Ti–C bonding features was examined by XPS measurements. A quantitative portion of ionic bonds in the composites was calculated with the area underneath the peaks for Ti–C ionic bonds, Ti–Ti metallic bonds, and C–C covalent bonds. Table S1 summarizes the peak area corresponding to each bond in the detected sample: Ti (2p_1/2_) and Ti (2p_3/2_) spin-orbital splitting photoelectrons to Ti^4+^ are identified at excitation energies of 463.5 and 457.9 eV; carbon bonds with the π‒π^*^ shake-up, sp^2^ hybridization are detected at binding energies of 286.3, and 283.4 eV, respectively; the binding energy of Ti‒C bonds is present at 282.8 and 454.5 eV for C 1s and Ti 2p, respectively. Table S2 shows the peak area of each bond (Ti‒C, C‒C and Ti‒Ti) normalized with the reinforcement volume (i.e., 1 vol. %). The Ti‒Ti bonds are the sum of Ti (2p_1/2_) and Ti (2p_3/2_) bonds. The C‒C bonds, on the other hand, are a sum of bonds with the π‒π^*^ shake-up and sp^2^ hybridization.

The volume fraction of a certain bond in the materials consisting of A and B (*X_vol_*, A–B) can be calculated with the Avogadro number (*N*), atomic mass (*M*), density (*ρ*), unit cell parameter (*a*) and peak area (*A*, shown in Table S1) of the XPS spectra as follow [9]:

(S1)

The volume fractions for each Ti‒Ti, C‒Ti, Ti‒C, and C‒C bonds are calculated for the MWCNT/Ti and FLG/Ti composites, (see Table S2). Based on the calculations, the fraction of Ti–C bonds is calculated as follows:

(S2)

where *X_C-C_*, *X_C-Ti_, X_Ti-C_* and *X_Ti-Ti_* are volume fractions of C–C, C–Ti, Ti–C and Ti–Ti bonds, respectively. Each bonds are normalized by volume fraction of the reinforcement (1 vol. %). Other possible bonds (e.g., Ti‒O‒C bonds) are neglected for the sake of simplification. Such endeavors may result in errors in calculations. Despite the assumptions and simplifications made for the calculation, it is expected to be useful for qualitative comparison of bonding features in the MWCNT/Ti, and FLG/Ti composites.

**Table S1.** Summary of the binding energy and relative peak area of each bond in XPS spectra, see Fig. 2.

|  | **Ti 2p** | | | | | | | **C 1s** | | | | |
| --- | --- | --- | --- | --- | --- | --- | --- | --- | --- | --- | --- | --- |
|  | **BE (eV)** | | | | | **Area (E+4)** | | **BE (eV)** | | | **Area (E+4)** | |
|  | **Ti_1/2_** | | **Ti_3/2_** | | **Ti–C** | ***A_Al-Al_*** | ***A_Ti-C_*** | **C-C sp^2^** | **C-C _π−π_** | **C–Ti** | ***A_C-C_*** | ***A_C-Ti_*** |
| **MWCNT/Ti** | 464.5 | 459.76 | 457.59 | 454.83 | 456.63 | 36.72 | 3.23 | 283.56 | 282.16 | 286.12 | 0.89 | 0.54 |
| **FLG/Ti** | 464.06 | 459.95 | 456.8 | 458.29 | 454.89 | 35.58 | 7.34 | 283.47 | 282.37 | 286.75 | 1.25 | 0.53 |

**Table S2.** The volume fraction of bond phases for normalized 1 vol.% from XPS spectra.

|  | | **MWCNT/Ti** | **FLG/Ti** |
| --- | --- | --- | --- |
| ***X_vol.C.-C_*** | | 2.42 E+12 | 1.43 E+13 |
| ***X_vol.C-Ti_*** | | 1.18 E+11 | 13.7 E+12 |
| ***X_vol.Ti-C_*** | | 1.84 E+14 | 9.58 E+14 |
| ***X _vol.Ti-Ti_*** | | 1.83 E+15 | 1.75 E+15 |
| **Bonding fraction** |  | 19.0 | 35.2 |

**Table S3.** The values of ***a*** *and* ***b*** for Ti-based composites from XPS spectra.

|  | **MWCNT/Ti** | **FLG/Ti** |
| --- | --- | --- |
| ***a*** | 0.6475 | 0.4685 |
| ***b*** | 0.3525 | 0.5315 |

**C. Mechanical properties of C/Ti, C/Al composites**

The compressive properties of the specimens were evaluated using an Instron-type machine under a constant crosshead speed condition of an initial strain rate of 10^-4^ s^-1^ at room temperature. Rectangular specimens with a height-to-width ratio of 2:1 were prepared for compression tests. To minimize the effect of friction, two tungsten carbide plates (coated with a thin boron nitride film) were used to sandwich the compression specimens.

**(a) Ti‒based composites (b) Al‒based composites**

**Fig. S6.** Compressive stress‒strain curves of **(a)** Ti-based composites and **(b)** Al-based composites under compression as a function of volume fraction of reinforcement.

To explain the strengthening behaviors of the composites, the yield stress is employed as a function of the volume fraction of reinforcements. Fig. S6 shows the compressive stress–strain curves of C/Ti and C/Al composites with various volume fractions of reinforcements. Table S4 compares the composites in this study with Al and Ti matrix composites reinforced with graphene or MWCNTs, which have recently been synthesized using various powder metallurgy (PM) routes [10-14]. The composites in this study exhibit world-record strength in comparison with previous reports, even though they were produced from cheap carbon materials via an industrially favorable route (simple hot-pressing). According to the results, *dE_c_/dV_f_* of Ti-based composites is about 5 times higher than that of Al-based composites, which corresponds roughly to the results from the simulations as well [24, 25]. Moreover, *dσ_c_/dV_f_* of Ti-based composites is 7.5 times higher than that of Al-based composites; *dσ_c_/dV_f_* of MWCNT-reinforced composites is 5.5 times higher than that of FLG-reinforced composites for both Ti and Al matrix.

Nonetheless, a selection of proper mechanical dispersion processing conditions may allow the uniform dispersion of nano-C materials within metal powders, providing high-quality composite samples with limited defects and porosity.

**Table S4**. Summary of previous works on metal/nano-C composites

| **Composite** | **Fabrication techniques** | **Yield stress**  **(MPa)** | **Research group** |
| --- | --- | --- | --- |
| Al/0.3 wt% Graphene | Ball-milling  Sintering : 580 ^o^C, 2h  Hot extrusion : 440 ^o^C, 20:1 | 250 | Wang et al. [10] |
| Al/0.1 wt% Graphene | Ball-milling  Hot isostatic pressing : 550 ^o^C, 4h  Hot extrusion : 550 ^o^C, 4:1 ratio | 262 | Bartolucci et al. [11] |
| Al/0.1 wt% Graphene | Ball-milling  Sintering : 600 ^o^C, 6h  Hot extrusion : 470 ^o^C, 2:1 ratio | 280 | Rashad  et al. [12] |
| Al/0.5 wt% Graphene | Ball-milling  Hot pressing : 500 ^o^C, 1h | 450 | Present study |
| Ti/0.4 wt% Graphene | Ball-milling  Spark plasma sintering : 800 ^o^C  Hot extrusion : 1000 ^o^C, 6:1 | 505 | Li et al. [13] |
| Ti/0.3 wt% Graphene | Ball-milling  Hot pressing : 570 ^o^C, 1h | 1450 | Present study |
| Ti/0.35 wt% MWCNT | Ball-milling  Sintering : 600 ^o^C  Hot extrusion : 440 ^o^C, 6:1 | 697 | Kondoh  et al. [14] |
| Ti/0.4 wt% MWCNT | Ball-milling  Spark plasma sintering : 800 ^o^C  Hot extrusion : 1000 ^o^C, 6:1 | 532 | Li et al. [13] |
| Ti/1.5 wt%  MWCNT | Ball-milling  Hot pressing : 570 ^o^C, 1h | 1270 | Present study |

On the other hand, the strengthening efficiency is drastically decreased with large contents of the reinforcement. The composites containing high contents of the reinforcement tend to aggregate. Figure S6 shows SEM images of Al-based composite powders containing 1.4 vol% FLG or 10 vol% MWCNT. FLG or MWCNT clusters are observed on the powder surface for composites with high contents of nano-carbon materials, whereas most nano-carbon materials are individually dispersed and embedded within the powder in composites with low contents of nano-carbon materials. These carbon aggregates may restrict consolidation of the composite powder, generating pores or carbides in the final composite, and thereby deteriorating the mechanical properties of the final composites.


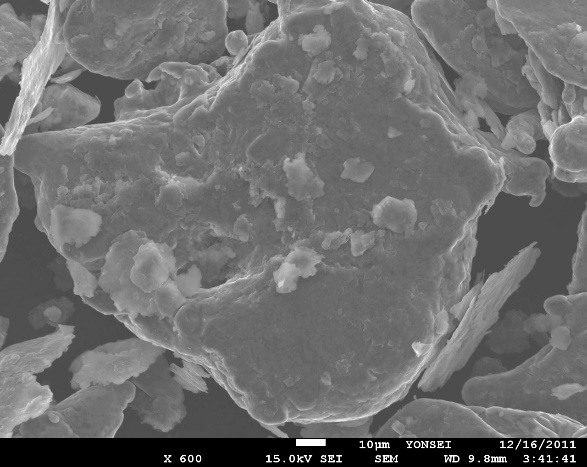

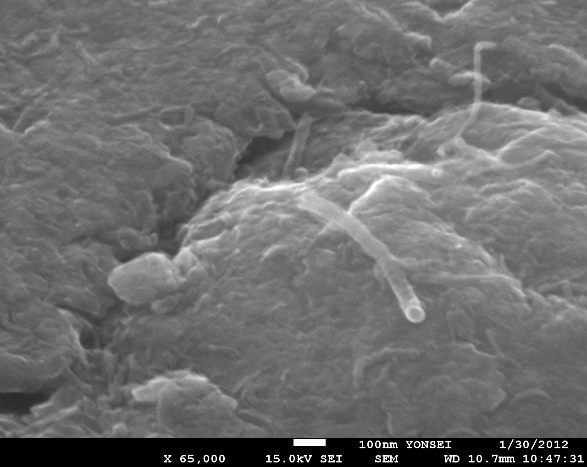


**(a)**

**(b)**

**FLG**

**MWCNT**

**50 μm**

**100 nm**

**(c)**

**Fig. S7.** SEM images of (a) Al/FLG and (b) Al/MWCNT composite powders. (c) Yield stress as a function of the volume fraction of reinforcements (FLG and MWCNT).

**D. Strengthening models**

Strengthening in the nano-carbon/metal composite can be described by a couple of mechanisms including grain boundary strengthening (e.g., Hall-Petch strengthening [15]), dispersion strengthening (e.g., Orowan strengthening [16]) and composite strengthening (based on load transfer from matrix to reinforcement, e.g., shear-lag theory).

The Hall–Petch relation calculates the effect of the grain size (*d*) on the yield stress (*σ_y_*), expressed as [16];

*σ_y_ = σ_o_ + Kd^-(1/2)^* (S3)

where *σ_o_* and *K* are empirical constants; *σ_o_* is the intrinsic stress resisting dislocation motion in a lattice and *K* is a measure of the resistance to dislocation motion caused by the presence of grain boundaries. The physical meaning is that greater stresses can be concentrated near the adjacent grains when the grain size is relatively large owing to the presence of multiple pile-up dislocations, leading to decreased yield stress. As shown in Figs. S8(a) and (b), ), the Al-based and Ti-based composites in this study had grain sizes of ~250 and ~300 nm, respectively, regardless of the volume or type of nano-carbon material. Such small volumes of nano-carbon materials are thought to rarely affect the grain size of the matrix. The strengthening of the matrix (ultrafine-grained monolithic Al and Ti) can be calculated using the Hall–Petch relation. The yield strength calculated using Eq. (S3) is comparable with the experimentally measured value for both Al and Ti. The yield stress was found to increase approximately 180 and 240 MPa by refining the grain sizes of Al and Ti, respectively.

**500 nm**


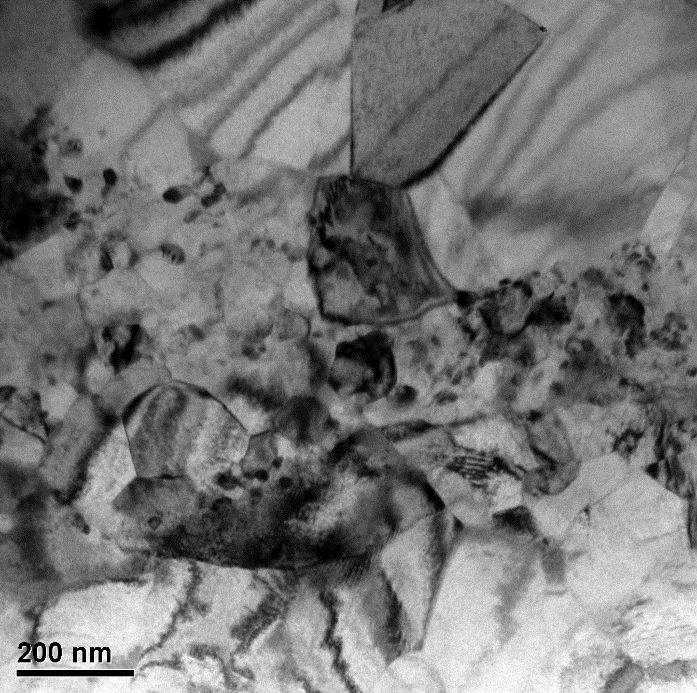

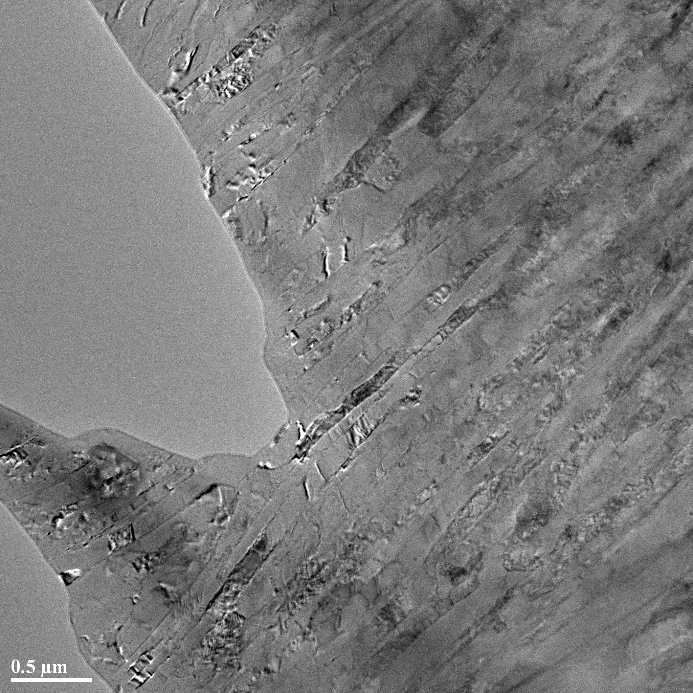


**500 nm**

**(a)**

**200 nm**

**(b)**

**Fig. S8.** HRTEM images of the (a) Al/FLG and (b) Ti/FLG composites.

In addition, Orowan strengthening assumes that the strengthening by restricting activities of dislocations to bypass the reinforcement while this model is valid only when the matrix plastically deforms and the reinforcement is located in grain interiors (rather than at grain boundaries). The reinforcement in this study (i.e., multi-walled carbon nanotube (MWCNT) and few-layered graphene (FLG)) is mostly located at the grain boundaries (as shown in Fig. S9) because of their high aspect ratio. It should be noted that the mean grain size of the matrix (250 and 300 nm for Al and Ti, respectively) is smaller than the length of the reinforcement. Therefore, active interactions between dislocations and the reinforcement are not expected during plastic deformation, and we consider Orowan strengthening to be minimal compared with other strengthening mechanisms.


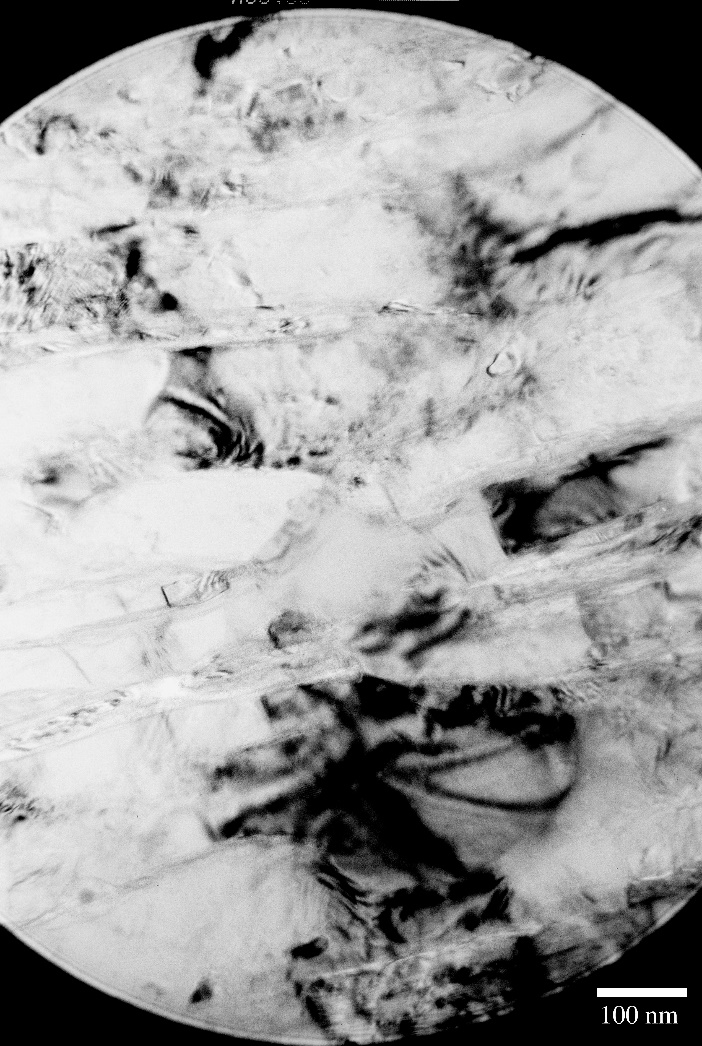


**100 nm**

**(a)**


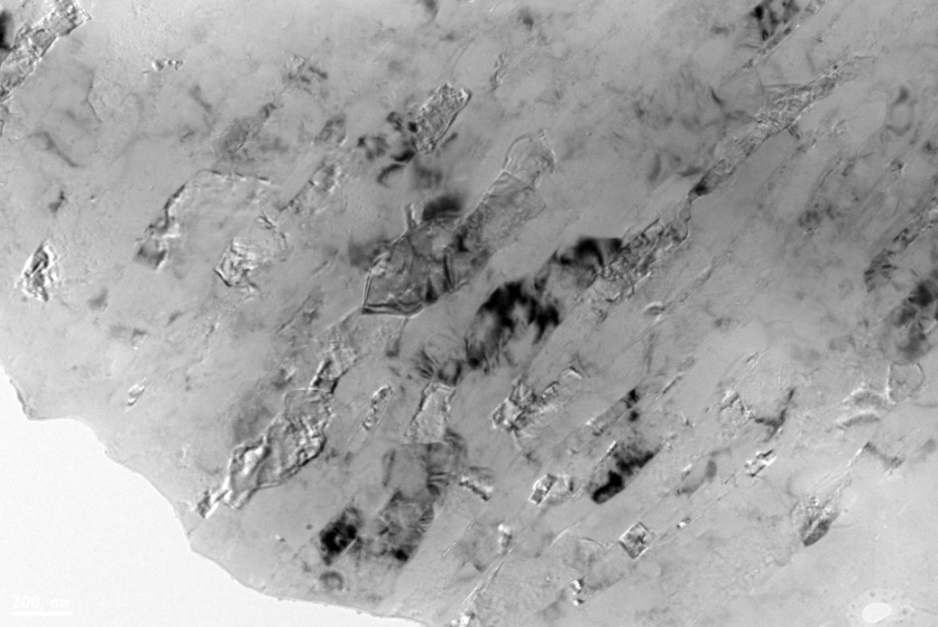


**200 nm**

**(b)**

**Fig. S9.** HRTEM images of Al/MWCNT and Al/FLG composites where the MWNCTs and FLG are marked by arrows.

Thus, the strengthening of the MMNCs is relatively difficult to predict precisely because it is influenced by several factors including load-transfer and interfacial bonding of the composites. Hence, we consider that the dominant strengthening mechanism of nano-carbon/metal composites in this study is composite strengthening, based on the load transfer from the matrix to the discontinuous reinforcement. Although several models (e.g., short-fiber model (namely, shear-lag theory) [15] and modified shear-lag theories [17]) have been suggested to describe the composite strengthening by discontinuous reinforcement, they all are derived from the same force balance theory. They assume that the discontinuous reinforcement is embedded in an elastic matrix with a perfectly bonded interface and uniaxial aligned to the loading direction. When tensile load is applied to composites containing discontinuous reinforcements (e.g., MWCNT and FLG), the strained matrix transfers load to the reinforcements by shear forces. These forces are generated between the reinforcement and the matrix. We start with a model to describe the strengthening by fiber-type reinforcement. Under the equal-strain condition (*ε_c_= ε_r_ = ε_m_ ,* where* ε_c_*, *ε_r_* and *ε_m_* are the strain of the composite, reinforcement and the matrix, respectively), a force balance forms the following equation [15]:

(S4)

where *τ_m_* is the shear strength of the matrix (~ 0.5* σ_m_*, *σ_m_* is the yield strength of the matrix). This also apparently denotes the shear stress that the matrix transmits to the fiber.
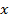
 is the axial distance from the tip of the fiber while *d_f_* is the average diameter of the fiber. Lastly, *σ_f_* is the position specific stress of the fiber (30 GPa for MWCNT [18]). Maximum stress carried by the fiber occurs at its midpoint; it reaches fiber strength when the fiber length equals to the critical length (*l_c_*). This can be expressed as [15]:

(S5)

Considering shear stress at the interface to be constant, for *l > l_c_*, the tensile strength of the composites (*σ_c_*) can be expressed as:

(S6)

where *V_m_* and *V_f_* are the volume fraction of the matrix and the reinforcement, respectively.

(S7)

For *l < l_c_*, the maximum stress in the reinforcement may never reach *σ_f_*. In this case, the reinforcement is not fractured, and instead the composite eventually fails by matrix tensile failure. By taking critical reinforcement length into account, the strength of the composite can be expressed as follows:

(S9)

Nardone and Prewo modified the shear-lag theory [17] for rectangular plate reinforcements as follows:

(S10)

where *L* is the length of the plate edge parallel to the applied stress and *t* is the thickness of the plate. By applying a simplification to the reinforcements, this group previously proposed a modified model that can be used for composites reinforced by both fibers and platelets [11]:

(S11)

where *S* is the surface area and *A* is the cross-sectional area of the reinforcement. As compared in Fig. S10, the experimental data of Al/FLG composites (Fig. S6 (a)) are well-matched with the modified shear-lag model (Eq. (S10) and our model Eq.(S11)), whereas those of the Al/MWCNT composite (Fig. S6 (b)) are in good accordance with the conventional shear-lag model (Eq. (S9) and our model Eq. (S11)).

(a)

(b)

(c)

(d)

**Fig. S10.** The variation of yield stress of (a), (b) Al-based and (c), (d) Ti-based composites with increasing the volume fraction of the reinforcements (FLG and MWCNT), in which the experimental data are compared with the theoretical expectations based on short-fiber model (shear-lag model), modified shear-lag model and the new model in this study.

However, the experimental data of Ti-based composites deviate significantly from theoretical expectations. This deviation has three possible origins: i) orientation, ii) interface, and iii) size effect. The reinforcement might not be perfectly aligned to the loading direction, and the misorientation of the reinforcement may decrease the strengthening efficiency. Also, the reinforcement might have weak bonding with the matrix, the strengthening efficiency is deteriorated.

The interfacial bonding between the reinforcements and the matrix (the bonding factor, *k*), the geometry of the reinforcement considering the surface-to-volume ratio (the geometry factor, g), alignment of reinforcements (the alignment factor, *s*) is the key parameters in nanocomposites. We have reconstructed equation to emphasize the effects of the three parameters to the load transfer behavior of a discontinuous reinforcement.

The average binding energy at the metal-carbon (M–C) interface can be calculated by the rule of mixture of M–C van der Waals bonds and M–C ionic bonds. The calculated average energy is normalized with the M–C ionic bonding energy. Subsequently, *k* becomes 1 as the matrix and the reinforcement form perfect tight bonds (i.e., 100% ionic bonds). This is expressed as [9]:

(S12)

where
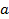
 and
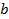
 are the volume fraction of M‒C van der Waals and M–C ionic bonds. These are evaluated from the XPS analysis. The calculation was carried out assuming that an obvious contrast between the Ti and Al matrix composites is distinction of the volume fraction of van der Waals and ionic bonds of M‒C composition. The sum of van der Waals and ionic bonds presents the total bonds, which is influential bonds, quantitative analysis for volume fraction of ionic bonds obtained from the XPS analysis. The results are comparable to the theoretical and experimental works as shown in figure 2 (The values of ***a*** and ***b*** are shown in Table S3). Moreover, ***E_a,M-C_*** (2.7 eV for Ti–C and 0.54 eV for Al–C) and ***E_b,M-C_*** (5.4 eV for Ti–C and 4.4 eV for Al–C) are Van der Waals and ionic bonding energy of M‒C bonds, respectively. The geometry factor, *g*, is defined as the aspect ratio and surface area of the reinforcement per unit volume. The surface area can be controlled by interfacial area (*S*) and the cross-sectional area (*A*) of reinforcements. It is worth noticing that, for mathematical accordance, the factor *g* is defined as the *S*-to-*A* ratio rather than the length-to-diameter ratio (that is, aspect ratio), and the volume-to-surface area of reinforcements.

On the other hand, another important aspect is that effect of nano-sized reinforcement on the strengthening efficiency. For instance, when the strength predicted by previous models, strengthening efficiency of two reinforcements with same aspect ratio is the same although their sizes are different. Indeed, researchers have developed a number of modified models to consider the effect of the orientation and interface of the reinforcement. For example, some approaches have introduced the concept of “effective aspect ratio” to consider the orientation between the reinforcement and the loading direction [20]. Moreover, several approaches have employed the concept of “interface layer” to account for the effect of interfacial bonding between the reinforcement and the matrix [21]. However, these deviations (misorientation or weak interface) from the ideal case should reduce the strengthening efficiency of the reinforcement, whereas the strengthening efficiency of FLG in Ti is positively deviated from the theoretical expectations. On the other hand, some experimental studies [22, 23] have also reported an increase of strengthening efficiency with decreasing reinforcement size, although the aspect ratio of the reinforcement does not vary. These observations cannot be explained by conventional load-transfer theories. The yield stress of the composites has also been observed to increase proportionally with the surface area per unit volume, regardless of the type of reinforcement [19]. Hence, rather than an aspect ratio (formulated by the surface-to-cross sectional area ratio, *S*/*A*), we hypothesize that a size factor (formulated by the volume-to-surface ratio) should be considered as a geometry factor to clarify the enhanced strengthening efficiency. Furthermore, the total number of bonds between metal and carbon atoms is also relevant, which with the volume-to-surface ratio consequently determines the interfacial features.

With this scope, we modified the rule of mixture using numerical efficiency parameters; (i) interfacial bonding, ***k*** (ii) geometry, ***g*** and (iii) alignment, ***s****.* Under the equal-strain condition, the elastic modulus of composites can be calculated by the simple rule of mixtures. This rule predicts the upper limit of the elastic modulus of the composite [15]:

(S11)

where *E_c_*, *E_m_* and *E_f_* are the elastic modulus of composite, matrix, and the reinforcement respectively. The elastic modulus for the particle-reinforced materials can be expressed as [15]:

(S12)

where *K* is an empirically determined “reinforcement factor”, which depends on the load transfer efficiency between the matrix and the reinforcement. Here, we specify the equations to estimate the mechanical properties (elastic modulus and strength of the composite) by substituting efficiency factor (***f***), which
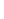
is the product of ***k, g*** and ***s*** expressed as:

(0 < *k, g, s* < 1) (S13)

Note that, in Eq. (S13), the geometry factor*, g,* takes account for the scale factor (expressed by volume-to-surface area ratio) as well as the aspect ratio (expressed by surface-to-cross sectional area ratio).

Finally, we modified the rule of mixture with these parameters as follows:

(S14)

(S15)

where *E_r_* is the elastic modulus of the reinforcement, *V_r_* is the volume fraction of the reinforcement and *σ_r_* is the yield strength of the reinforcement, respectively. Dominant strengthening mechanism of MMNC is load transfer. Hence, on the assumption that load transfer can be totally used to contribute strengthen the nanocomposites, elastic modulus and strength of the composites proportionally increases with the specific surface area per unit volume regardless of the type of the reinforcement (Fig. 3).

According to plots (Fig.3), the values of *c* (for elastic modulus) and *c'* (for strength) are 33.8 and 14.4, respectively. These constants are normalized values, which are calculated by dividing the slope of increment of elastic modulus and strength by factors for the geometry (g, size factor and aspect ratio), orientation (s, alignment) and interfacial bonding (k, ionic and/or covalent bonding) of the reinforcement as noted in Eqs. (R10)-(R12). *k* and *s* are strongly affected to the matrix, and *g* is related to characteristic of the reinforcement, therefore the factors are separately positioned.

Using the Eq. (R11) for elastic modulus can be expressed as:

(S16)

Also, using the Eq. (S15) for strength can be expressed as:

(S17)

The values are offset by orientation and interfacial bonding factors and hence the remained value is determined only by the reinforcement itself (i.e., nano-C in this study). Therefore, each terms of the left side and the right side can be understand for variable of the composite and the reinforcement, respectively. According to Eqs. (S16) and (S17), reciprocal values of *1/c* ~ 0.03 and *1/c'* ~ 0.07 can be determined. The calculated values of 3 and 7% for the elastic modulus and strength, respectively, stand for total-factor energy efficiency used for strengthening nanocomposites.

The results provide a guideline for the design of MMNCs with diverse reinforcements using the reliable model to predict mechanical properties of MMNCs.

**References**

[1] Tokunaga T., Kaneko K., Horita Z. & Iijima S. Production of aluminum-matrix carbon nanotube composite using high pressure torsion. *Mater. Sci. Eng. A.* **490**, 300–304 (2008).

[2] Castiglioni C., Tommasini M. & Zerbi G. Raman spectroscopy of polyconjugated molecules and materials: confinement effect in one and two dimensions. *Phil. Trans. R. Soc. Lond. A.* **362,** 2425–2459 (2004).

[3] Kudin K.N., Ozbas B., Schniepp H.C., Prud’homme R.K., Aksay I.A. & Car R. Raman Spectra of Graphite Oxide and Functionalized Graphene Sheets. *Nano. Lett.* **8**, 36–41 (2008).

[4] Tarantili P.A & Andreopoulos G.C. Real-time micro-raman measurements on stressed polyethylene fibers. 1. Strain rate effects and molecular stress redistribution. Macromolecules 31,6964–6976 (1998).

[5] Wang Z., Ciselli P. & Peijs T.. The extraordinary reinforcing efficiency of single-walled carbon nanotubes in oriented poly(vinyl alcohol) tapes. *Nanotechnology* **18**, 455709 (2007).

[6] Antunes E.F., Lobo A.O., Corat E.J., Trava-Airoldi V.J., Martin A.A. & Veríssimo C. Comparative study of first- and second-order Raman spectra of MWCNT at visible and infrared laser excitation. *Carbon* **44**, 2202–2011 (2006).

[7] Ferrari A.C. et al., Raman spectrum of graphene and graphene layers. *Phys. Rev. Lett.* 2006;97(18):187401.

[8] Li D. et al., Thickness and stacking geometry effects on high frequency overtone and combination Raman modes of graphene. *J Raman Spectrosc* **44**,86–91 (2013).

[9] Lewin E et. al.,. On the origin of a third spectral component of C1s XPS-spectra for nc-TiC/a-C nanocomposite thin films. *Surf. Coat. Tech.* **202**, 3563–3570 (2008).

[10] Bartolucci S.F. et al. Graphene-aluminum nanocomposites. *Mater. Sci. Eng. A.* **528**, 7933–7937(2011).

[11] Wang, J., Li, Z., Fan, G., Pan, H., Chen, Z. & Zhang, D. Reinforcement with graphene nanosheets in aluminum matrix composites. *Scripta. Mater.* **66**, 594–597 (2012).

[12] Rashad M, Pan F, Tang A & Asif M. Effect of Graphene Nanoplatelets addition on mechanical properties of pure aluminum using a semi-powder method. *Prog. Nat. Sci. Mat. Int.* **24**, 101–108 (2014).

[13] Li, S., Sun, B., Imai, H., Mimoto, T., & Kondoh, K. Powder metallurgy titanium metal matrix composites reinforced with carbon nanotubes and graphite. *Comp. Part. A.* **48**, 57–66 (2013).

[14] Kondoh, K., Thotsaphon, T., Imai, H., Umeda, J. & Fugetsu, B. Characteristics of powder metallurgy pure titanium matrix composite reinforced with multi-wall carbon nanotubes. *Comp. Sci. Technol.* **69**, 1077–1081 (2009).

[15] Courtney T.H. Mechanical Behavior of Materials 2nd edn, (McGraw-Hill Book Co., 2000).

[16] Zhang, Z. & Chen, D.L. Consideration of Orowan strengthening effect in particulate-reinforced metal matrix nanocomposites: A model for predicting their yield strength. *Scripta. Mater.* **54** 1321-1326 (2006).

[17] Nardone, V.C., & Prewo, K.M. On the strength of discontinuous silicon carbide reinforced aluminum composites. *Scripta. Metall. Mater.* **20** 43-48 (1986).

[18] Zhong R., Cong H. & Hou P. Fabrication of nano-Al based composites reinforced by single-walled carbon nanotubes. *Carbon* **41,** 848–851 (2003).

[19] Shin, S.E., Choi, H.J., Shin J.H. & Bae, D.H. Strengthening behavior of few-layered graphene/aluminum composites*. Carbon*. 82, 143–151 (2015).

[20] Rashid, K. & Abu, A. Modeling the interfacial effect on the yield strength and flow stress of thin metal films on substrates. *Mech. Res. Commun.* **35** 151-157 (2008).

[21] Starink, M.J. & Syngellakis, S. Shear lag models for discontinuous composites: fibre end stresses and weak interface layers *Mater. Sci. Eng. A.* **270**, 270-277 (1999).

[22] Zhao, Q. & HOA, S.V. Toughening Mechanism of Epoxy Resins with Micro/Nano Particles. *J. Comp. Mater*.**41**, 201-219 (2007).

[23] Rashid K. Abu Al-Rub. Interfacial gradient plasticity governs scale-dependent yield strength and strain hardening rates in micro/nano structured metals. *Int. J. Plasticity*. **24**, 1277-1306 (2008).

[24] Nakada, K. & Ishii A. DFT calculation for adatom adsorption on graphene sheet as a prototype of carbon nano tube functionalization. *J. Phy. Conf. Ser.* **100,** 052087 (2008).

[25] Park, N., Sung, D., Lim, S., Moon, S. & Hong, S. Realistic adsorption geometries and binding affinities of metal nanoparticles onto the surface of carbon nanotubes. *Appl. Phys. Lett.* **94,** 073105-1‒3 (2009).

1. ^^ Corresponding author (Donghyun Bae) Tel.:+82 2 2123 5831; Fax:+82 2 312 5375

   E-mail address: [donghyun@yonsei.ac.kr](mailto:donghyun@yonsei.ac.kr) [↑](#footnote-ref-2)
